# Epigenetics in cancer therapy and nanomedicine

**DOI:** 10.1186/s13148-019-0675-4

**Published:** 2019-05-16

**Authors:** Annalisa Roberti, Adolfo F. Valdes, Ramón Torrecillas, Mario F. Fraga, Agustin F. Fernandez

**Affiliations:** 1 Cancer Epigenetics Laboratory, Institute of Oncology of Asturias (IUOPA), ISPA-FINBA-Hospital Universitario Central de Asturias HUCA, Universidad de Oviedo, Avenida de Roma, 33011 Oviedo, Asturias Spain; 2 Nanomaterials and Nanotechnology Research Center (CINN-CSIC)-Universidad de Oviedo-Principado de Asturias, Avenida de Roma, 33011 Oviedo, Asturias Spain

**Keywords:** Epigenetics, DNMT inhibitors, HDCA inhibitors, Nanomedicine, Nanoparticles, Nanocarriers

## Abstract

The emergence of nanotechnology applied to medicine has revolutionized the treatment of human cancer. As in the case of classic drugs for the treatment of cancer, epigenetic drugs have evolved in terms of their specificity and efficiency, especially because of the possibility of using more effective transport and delivery systems. The use of nanoparticles (NPs) in oncology management offers promising advantages in terms of the efficacy of cancer treatments, but it is still unclear how these NPs may be affecting the epigenome such that safe routine use is ensured. In this work, we summarize the importance of the epigenetic alterations identified in human cancer, which have led to the appearance of biomarkers or epigenetic drugs in precision medicine, and we describe the transport and release systems of the epigenetic drugs that have been developed to date.

## Introduction: epigenetics

Although all cells of a body have essentially the same genes, it is the epigenetic information which regulates how the genome is read and manifests itself across different developmental stages and in cellular differentiation and lineage commitment in adult tissues. This epigenetic information is stored as covalent modifications of chromatin components which, by transforming the local chromatin environment, affect DNA accessibility and provide docking/recognition sites for regulatory protein binding, thus influencing the transcription and function of a gene without affecting the nucleotide sequence of the gene itself.

Epigenetic modifications are relatively mitotically and/or meiotically heritable, allowing the transfer of gene function information from one cell generation to the next in order to guarantee that cellular identity and lineage fidelity are preserved. However, although in the case of DNA methylation mitotic inheritance is well proven, the situation does not seem so clear in the case of some post-translational modifications such as histone acetylation [1–5]. Importantly, epigenetic modifications are reset in primordial germ cells (PGCs), the precursors of sperm and oocytes, preparing them for development in future generations [6]. Since epigenetic marks do not change genetic material, this epigenetic reprogramming ensures that genomic information, inherited from parents, remains untouched.

**Fig. 1:**
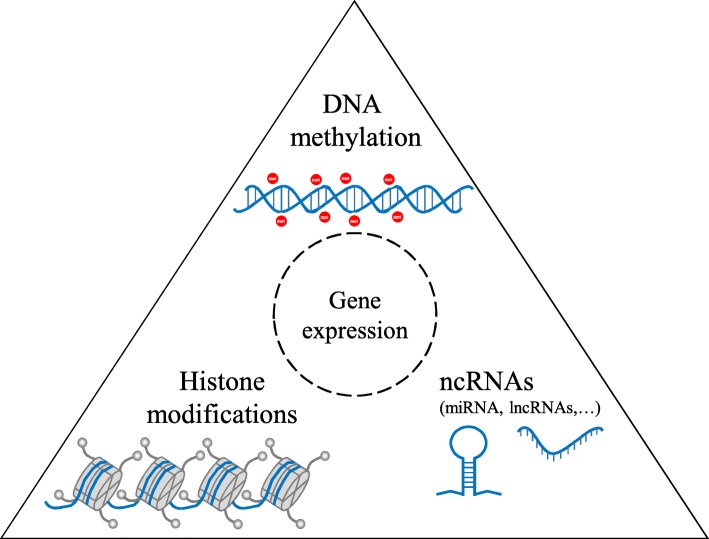
Epigenetic mechanisms contributing to gene regulation. DNA methylation, histone modifications, and noncoding RNAs (ncRNAs)

The epigenetic machinery is composed principally of three interconnected components: DNA methylation, histone post-translational modifications, and non-coding RNAs (ncRNAs) (Fig. 1). DNA methylation, has been recognized as a key regulatory mechanism during development, cellular differentiation, and tissue homeostasis. It has been associated with several physiological and pathological processes, including genomic imprinting, X chromosome inactivation, tissue-specific gene expression, chromosome stability, repression of transposable elements, aging, and a number of diseases, including cancer [2, 7]. DNA methylation is defined as the covalent transfer of a methyl group to the C-5 position of the cytosine ring of DNA, which is catalyzed by DNA methyltransferases (DNMTs). Three enzymes are involved in the generation and maintenance of DNA methylation patterns. DNMT1, defined as the maintenance methyltransferase, has a strong preference for hemi-methylated CpG dinucleotides, thus can methylate CpG in a newly synthesized DNA strand based on the presence of methylation in the complementary template. DNMT3A and DNMT3B show no preference for hemi-methylated target sites and are instead involved in de novo DNA methylation, as well as in introducing non-CpG methylation. During mammalian development, DNA methylation can be passively lost or else actively driven by an enzymatic process involving the ten-eleven translocation (TET) family of dioxygenases which catalyzes the oxidation of methylcytosine to hydroxymethylcytosine, which is followed by glycosylation and replacement with an unmethylated cytosine [8].

In mammals, DNA methylation occurs almost exclusively in the context of CpG dinucleotides, and genomes are globally CpG-depleted and generally methylated. Less than 10% of total CpGs are found at CpG islands, defined as unmethylated GC-rich regions that possess high relative densities of CpG and are positioned at the 5′ ends of many human genes [9]. Although the great majority of CpG islands are unmethylated during all stages of development and in all tissue types, there is a subset which undergoes developmentally programmed methylation. The process of de novo methylation is not only active in germ cells or in early embryo stages, but it can also occur in adult somatic cells, as demonstrated by the progressive methylation of a fraction of human CpG islands during aging [10] or in abnormal cells [9, 11]. When located in a gene promoter, DNA methylation usually marks genes for transcriptional silencing. Mechanistically, a methylated cytosine can promote or preclude the recruitment of regulatory proteins, and this can, respectively, mediate transcriptional repression through interactions with histone deacetylase or exclude transcription factors or other transcriptional regulator components from their target sites [2].

Another form of epigenetic information, closely interconnected with the DNA methylation machinery, is the post-translational modification of nucleosomal histones (Fig. 1). Most of these post-translational marks occur at a specific position within amino-terminal- and carboxy-terminal histone tails, although modifications within the central domains of the histones have also been identified. Since chromatin is the physiological template of all genetic information, each modification can produce different functional consequences through influencing chromosome structure, thereby defining various functional domains of chromatin. Histones are subject to more than 200 known modifications, including acetylation, methylation, phosphorylation, ubiquitylation, sumoylation, and ribosylation. Adding to the complexity is the fact that each residue can accept one or more modifications and that the same mark or set of marks can have different effects depending on which residue is modified [12]. Numerous enzymes which direct histone modifications have been identified and characterized based on their specific activity and the residues modified. Most of the modifications are dynamic, and enzymes that remove the modification have been identified as well. Acetyltransferase and deacetylase coordinate histone acetylation; methyltransferases and demethylases control histone methylation and have high catalytic specificity; serine/threonine kinases and histone ubiquitin ligases promote respectively histone phosphorylation and ubiquitination. Enzymes responsible for histone sumoylation, ADP-ribosylation, deamination, and proline isomerization have also been characterized (for a detailed review, see [13]).

Regardless of which residue is modified, lysine (K) acetylation almost always correlates with chromatin accessibility and transcriptional activation. In contrast, lysine methylation has contrasting effects depending on which residue is modified. For example, methylation of H3K9, H3K27, and H4K20 generally correlates with repressed chromatin state, while methylation of H3K4 and H3K36 is associated with transcribed chromatin and gene activity [2, 12]. The phosphorylation of several serine and threonine residues on histones facilitates chromatin condensation during mitosis and transcriptional activation of immediate-early genes, and it has also been implicated in DNA repair and apoptosis [14–16]. One very well-characterized modification involves the phosphorylation of H2AX on serine 139 in the presence of DNA damage. This newly phosphorylated protein, termed γ-H2AX, is the first step in recruiting and localizing DNA repair proteins [17]. Furthermore, the ubiquitylation or sumoylation of certain specific residues has been linked to transcriptional repression mitosis, euchromatin, and spermatogenesis.

An additional layer of complexity is added by the interdependency between different modifications and the fact that adjacent modifications can influence each other. As mentioned above, each specific pattern of epigenetic modification is associated with a particular chromatin state and thus correlates with a specific biological function. However, in pluripotent cells, opposing histone modifications can co-localize the same region, known as a “bivalent domain.” In embryonic stem cells (ESCs), promoters of many genes are associated with both active H3K4me3 and repressive H3K27me3 chromatin marks. These domains play a pivotal role in maintaining pluripotency and keeping developmental genes silenced or expressed at very low levels in the absence of differentiation signals while at the same time keeping them poised for timely activation [18, 19]. Growing evidence shows that bivalent chromatin is not unique to ESCs, but rather this euchromatin/heterochromatin border state could be what is responsible for priming the rapid gene expression changes in T cell activation upon antigen recognition and that disturbance of bivalent chromatin may be intimately linked to tumorigenesis [20].

Although ncRNAs do not fully meet the classical definition of epigenetic regulators, their ability to regulate gene expression on the post-transcriptional and transcriptional level and to affect the organization and modification of chromatin both emphasize their role as epigenetic modulators (Fig. 1). They are a cluster of RNAs that are not translated in functional proteins, although they are nonetheless functional. Based on their size, ncRNAs can be divided into two main groups: short-chain non-coding RNAs (which includes siRNAs, miRNAs, and piRNAs) and long non-coding RNA (lncRNAs), having less or more than 200 nucleotides in length, respectively. Of the former, small interfering RNAs (siRNAs) and microRNAs (miRNAs) are similar in length (19–24 nucleotides) and biogenesis, and both are associated with translational repression and RNA cleavage through complementary pairing with mRNA target sites [21].

miRNAs are capable of controlling the expression of more than one RNA, a feature distinguishing them from siRNAs, which needs perfect complementarily for degradation. PIWI-interacting RNAs (piRNAs) are 26–30-nt-long, single-stranded endogenous ncRNAs mainly transcribed in germline cells. They are produced in clusters and then cleaved into individual units then bind to PIWI proteins to induce epigenetic regulation and transposon control. They play a critical role in guaranteeing genome integrity as well as in regulating post-transcriptional silencing of target RNAs (frequently transposable elements) through perfect or mismatched base pairing. It has been demonstrated that Piwi/piRNA complexes might serve as sequence-specific guides that direct the de novo DNA methylation machinery to transposable elements [22].

The second group, lncRNAs, refers to a heterogeneous class of RNA transcripts, including enhancer RNAs, small nucleolar RNA (snoRNA), intergenic transcripts, and transcripts overlapping other transcripts in either a sense or antisense orientation [23]. Together with their well-established role in X-chromosome inactivation, emerging evidence demonstrates that they play a role in numerous cellular processes, such as gene imprinting, differentiation, and development. lncRNAs can regulate transcription by interacting with chromatin-modifying complexes or with the transcriptional machinery. Furthermore, some lncRNAs act post-transcriptionally as regulators of splicing, mRNA stability, protein translation, and protein stability. IncRNAs do not only temporally and spatially modulate gene activity, but also regulate biological processes such as the DNA damage response, DNA repair, and DNA replication [24].

## Cancer epigenetics

The complexity of cancer biology can be explained as the interplay between genetic and epigenetic abnormalities that are mutually beneficial in order to drive cancer initiation and progression. The importance of epigenetic alterations as driving forces of tumor initiation clearly emerged from studies of pediatric cancers, especially brain tumors, which are characterized by few or no recurrent mutations, and are instead defined by their aberrant epigenetic patterns [25, 26].

Efforts to sequence the genome of thousands of human cancers over the past decade have elucidated the presence of frequent alterations in numerous epigenetic regulators, recognizing unambiguously the key role of epigenetic deregulation in carcinogenesis [27–30].

**Fig. 2:**
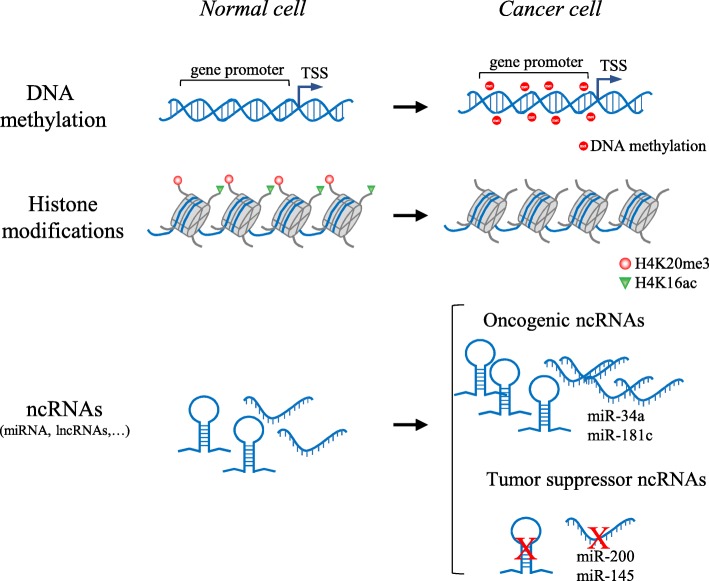
Examples of epigenetic alterations in cancer cells. Hypermethylation of promoters of tumor suppressor genes, global loss of H4K20me3 and H4K16ac, and up- or downregulation of miRNAs that target oncogenes and tumor suppressor genes, respectively

During tumorigenesis, the epigenome goes through multiple alterations, including genome-wide loss of DNA methylation and regional hypermethylation, especially in CpG promoter islands of tumor suppressor genes [31–34], global changes in histone modification marks [34–36], and deregulation in the networks in which ncRNAs engage [34, 37, 38] (Fig. 2).

High-throughput analysis of genome-wide DNA methylation has demonstrated that not only specific genes but also distinct epigenetic signatures are reproducibly found in nearly all cases of a specific type of cancer and that they specifically correlate with tumor stage and type [33, 39, 40]. These characteristics have encouraged the use of epigenetic signature as a potent biomarker for early detection, non-invasive screening, prognosis, and prediction of therapeutic response [39, 41–45]. An example of the usefulness of DNA methylation in clinical practice is the first epigenetic diagnostic test (EPICUP) based on the analysis of DNA methylation profiles to identify the primary tumor in patients with cancer of unknown origin (CUP). EPICUP is a diagnostic system, based on epigenetic profiling, that takes advantage of the specific DNA methylation patterns of each tumor type. CUP is a very aggressive type of cancer that generates metastasis even before the primary tumor becomes evident, despite standard test. Determining the primary tumor type is, however, central to initiating more accurate oncological treatment, which is associated with better outcomes. EPICUP has great specificity and sensitivity owing to it being based on DNA methylation profiles which classify CUPs with respect to samples of known origin, including 38 tumor types and 85 metastases [33, 45].

During tumorigenesis, cells also undergo global changes in histone modifications that contribute to aberrant gene expression [34–36]. It has also been demonstrated that histone-modifying enzymes have characteristic patterns of expression depending on the tissue of origin and can discriminate tumor samples from their normal counterparts and cluster the tumor samples according to cell type, indicating that abnormal expression of these proteins plays an important cancer-specific role in neoplastic transformation [46]. Although not as much progress has been made as in studies of DNA methylation, the identification of a specific histone mark signatures associated with each type of cancer is an important step not only in terms of more accurate diagnosis and prognosis, but also because it is fundamental to the design and evaluation of possible treatment with epigenetic drugs.

Moreover, genetic alterations in epigenetic enzymes seem to contribute significantly to the appearance of aberrant patterns of DNA methylation and/or histone modifications in cancer [29, 47].

The identification of mutations in members of the DNA methylation machinery serves as a starting point in the development of more precise diagnostic and prognostic tools in cancer management [47]. For example, *DNMT3A* is commonly mutated in acute myeloid leukemia (AML) and T cell lymphomas. Interestingly, it has been reported that DNMT3A mutations are present in hematopoietic stem cells (HSCs) from the blood of AML patients. These alterations confer enhanced self-renewal, leading to a clonally expanded pool of pre-leukemic HSCs, from which AML evolves by acquiring further mutations. These populations of cells are able to survive chemotherapy, and they might represent a reservoir from which relapse arises [48]. In addition, *TET2* mutations are an unfavorable prognostic factor in AML and are associated with increased response to hypomethylating agents in myelodysplastic syndrome (MDS) [49, 50].

Adult tumors, especially hematopoietic malignancies, are characterized by high-frequency mutations in gene encoding chromatin-regulating enzymes. The best-studied histone modification is the acetylation of lysine on histone tails, which is dynamically regulated by two enzyme families, histone lysine acetyltransferases (HATs) and histone deacetylases (HDACs). Numerous examples of translocations and mutations in HAT family members (p300, CBP, and MYSTA4) have been reported in both hematological malignancies and solid tumors. Germline mutations and overexpression of HDACs have been observed in various cancers, resulting in a global loss of histone acetylation and the consequent silencing of tumor suppressor genes [51].

SETD2 and MLL2, two genes that encode lysine methyltransferase, have also been found to be mutated in 93% of enteropathy-associated T cell lymphoma (EATL-II) and 89% of follicular lymphomas (FL), respectively, suggesting they might act as driver mutations in these tumors [52, 53]. SETD2-inactivating mutations have also been reported in renal cell carcinoma [54] and pediatric acute lymphoblastic leukemia, and they are correlated with poor outcome and disease relapse [55].

The histone modifier EZH2 (enhancer of zeste 2 polycomb repressive complex 2 subunit), which can function as either tumor suppressor gene or oncogene depending on the cancer type, is worthy of particular mention. It is the enzymatic subunit of polycomb repressive complex 2 (PRC2) which is involved in maintaining the transcriptional repressive state of genes through methylation on histone H3 lysine 27. EZH2 overexpression and gain-of-function mutations have been reported in several solid tumors (breast, ovarian, lung, liver, bladder, glioblastoma, etc.) and in non-Hodgkin’s lymphoma, concurrently with H3K27 trimethylation. On the other hand, recurrent inactivating deletions, frameshift, nonsense, and missense mutations in *EZH2* occur in a subset of MDS, myeloproliferative neoplasms (MPNs), and in human T cell acute lymphoblastic leukemia. Loss-of-function somatic alterations in genes encoding PRC2 subunits other than EZH2 also occur in tumors, and lysine residue 27 of histone H3 has itself been found to harbor specific recurrent missense mutations in highly restricted cancer types [56].

Taken together, these discoveries demonstrate how EZH2 gain- or loss-of-function mutations can promote the progression of cancer in a context-specific fashion, through increasing or decreasing H3K27 trimethylation levels, which in turn regulate specific patterns of gene expression.

Epigenetic machinery can also be deregulated indirectly by mutations in upstream effectors, i.e., epigenetic modulators. Heterozygous somatic mutations in the loci encoding isocitrate dehydrogenase 1 and 2 (IDH1/2) occur in ~ 20% of AMLs and are associated with global hypermethylation and gene-specific methylation signatures. These effects are caused in part through the inhibition of TET2. Interestingly, *IDH1/2* and TET2 mutations are mutually exclusive and biologically redundant. Mutated IDH1/2, but not the wild type, induces the inhibition of TET2 and, in turn, the alteration of gene expression though aberrant methylation. These observations demonstrate the way in which alterations in cellular metabolic pathways can lead to leukemic transformation through the dysregulation of the epigenetic machinery [57].

ncRNAs are also critical regulators of gene expression, and their deregulation has been associated with a growing number of cancers. Amplifications, deletions, and mutations can alter ncRNA expression and, as a result, are associated with the aberrant functioning of their specific targets. ncRNAs can have either an oncogenic or a tumor-suppressive function, or they can act in a context-dependent manner. In chronic lymphocytic leukemia (CLL), patients undergo a frequent deletion at the 13q14 region that encodes for miR-15 and miR-16, which are implicated in the apoptosis through their targeting of BCL2 [58]. The miR-17~92 cluster (13q31-q32) is amplified in diffuse large B cell lymphoma patients and acts with MYC to accelerate tumor development and is involved in chemo- and radio-resistance [59]. Although single miRNA can be either up- or downregulated, the overall miRNA expression is suppressed in tumor cells, as demonstrated by the documented alterations in miRNA processing machinery in several cancers. Ribonuclease III (RNase III) DROSHA and DICER1 have been shown to be downregulated in lung cancer, ovarian cancer, and neuroblastoma, and their level correlates with tumor stage and clinical outcome [60, 61].

The interconnection between ncRNAs and the epigenome makes the situation more complex still in that ncRNAs are able to influence gene expression by regulating the epigenetic machinery, while at the same time being themselves epigenetically regulated. In cancer, several ncRNAs, especially miRNAs, undergo transcriptional inactivation by promoter hypermethylation. This can result in the overactivation of their oncogenic targets.

DNA methylation-associated silencing of miR-34b/c, miR-148, and miR-9-3 is correlated with the loss of regulation of oncogenic target genes, such as C-MYC, E2F3, CDK6, and TGIF2, and with the activation of signaling that promotes invasiveness and metastasis [62].

The interconnectedness of epigenetics and ncRNAs has been demonstrated in lung cancer [63] as well as in AML [64] where the downregulation of the miR-29 family inversely correlates with DNMT expression and thus with aberrant DNA methylation. As a consequence, re-expression of miR-29 is able to restore the normal patterns of DNA methylation, to induce re-expression of methylation-silenced tumor suppressor genes, and to inhibit tumorigenicity. This discovery seems to provide pharmacological support for the use of synthetic miR-29b as an efficient therapeutic DNA hypomethylating agent [65]. miRNAs are known to regulate the expression of several components of Polycomb complexes, for example, downregulation of mi-101 is associated with the overexpression of the histone methyltransferase EZH2.

## Epigenetic approaches in cancer treatment

**Fig. 3:**
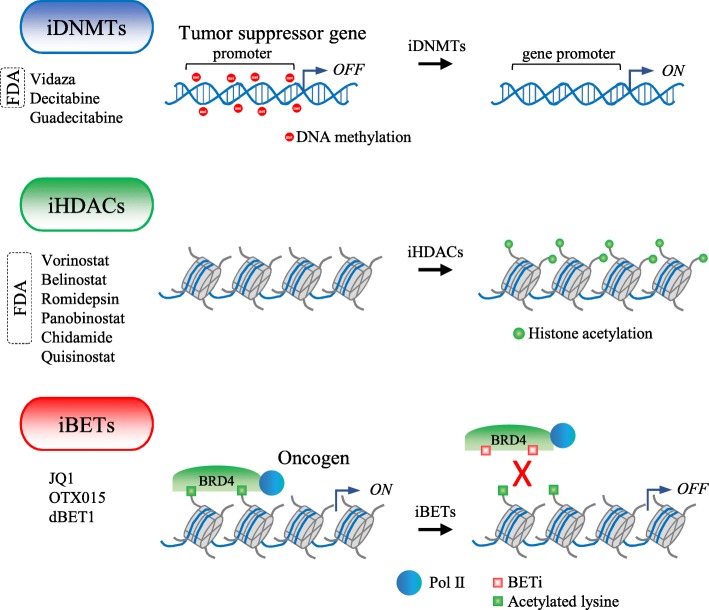
Examples of epigenetic drugs and their general mechanisms of action. Some of these drugs are approved by USFDA, and others are currently in clinical trials

Both the plasticity and the reversible nature of epigenetic modifications make them ideal potential druggable targets for anticancer strategies, the idea being that they enable the resetting of the cancer epigenome. Epidrugs can be classified on the basis of their respective target enzyme. Although at present only two classes of epigenetic drugs have been approved by the US Food and Drug Administration (FDA)—DNA methylation inhibitors (iDNMTs) and histone deacetylase inhibitors (iHDACs)—several new targets are in late-stage clinical trials and show therapeutic promise [31, 42, 47, 66] (Fig. 3).

The first approved epigenetic drug was 5-azacitidine (Vidaza, Azacitidine), a iDNMT indicated in the treatment of patients with MDS and AML, followed 2 years later in 2006 by 5-aza-2′-deoxycytidine (decitabine (DAC), Dacogen), which has the same clinical indication. At low doses, these cytosine analogs inhibit DNMT in actively replicating cells, causing the loss of methylation marks during DNA replication, and consequently the reactivation of aberrantly silenced tumor suppressor genes, which thus restores their expression and functional activity [67]. These inhibitors affect the key regulatory pathways such as apoptosis, cell cycle regulation, and immune modulation. Whereas DAC can in general only be incorporated into DNA strands, and azacitidine can be incorporated into both DNA and RNA chains. Although iDNMTs have been shown to be clinically efficacious, these drugs are not locus-specific and cause large-scale changes in gene expression, inducing not only the re-expression of genes that have been improperly silenced in cancer, but also the transcriptional activation of oncogenes and prometastatic genes [68].

Recent reports suggest that iDNMTs may exert their antitumoral activity not only through their hypomethylating action, but also by inducing double-strand DNA breaks and, consequently, G2 arrest [69], as well as by stimulating immune signaling through the viral defense pathway by means of inducing the reactivation of endogenous retroviral elements [70, 71].

HDACs remove acetylation marks from histone tails to establish a repressive chromatin environment. Several iHDACs are emerging as promising anti-cancer drugs that play important roles in epigenetic and non-epigenetic regulation, inducing cell death, apoptosis, cell cycle arrest, inhibition of cell mobility, and antiangiogenesis in transformed cells [31]. Their therapeutic effect is mainly mediated by the reactivation of abnormally silenced tumor suppressor genes (TSGs), although the mechanism is not yet fully understood. The USFDA has approved four iHDACs drugs. Vorinostat (SAHA), approved for the treatment of patients with cutaneous T cell lymphoma (CTCL), acts on class I, II, and IV HDACs and has been shown to induce apoptosis and cell cycle arrest, as well as to sensitize cancer cells to chemotherapy [72]. Belinostat selectively acts on class I and II HDACs and has been approved to treat peripheral T cell lymphomas (PTCL). Romidepsin specifically targets class I HDACs and has been approved for both CTCL and PTCL patients. The latest approved HDACi was panobinostat, indicated for the treatment of drug-resistant multiple myeloma in combination with proteasome inhibitor brotezomid. Panobinostat is the only HDACi approved in Europe for clinical use [73].

Although both iDNMTs and iHDACs are effective as single agents, their efficacy is enhanced in combinatorial therapy approaches, and, indeed, their most promising use, especially for solid tumors, may be in combination with each other as well as with other drugs [74–77]. Numerous trials have in fact demonstrated how combination therapy induces an enhanced upregulation of improperly silenced genes and produces a stronger antitumor effect as compared with single-agent treatment [31]. The synergistic effect of combining iDNMTs and iHDACs relies on the fact that HDAC-mediated histone deacetylation generally collaborates with DNA methylation in generating a transcriptionally repressive chromatin conformation.

Although promising data are emerging from a group of patients with non-small cell lung cancer (NSCLC) who have shown robust and lasting positive responses when treated with low doses of iDNMTs and iHDACs [76], conflicting results are emerging concerning the efficacy of iDNMT and iHDAC combination therapy in MDS and AML, i.e., smaller trials have shown increased efficacy, but recent large trials have shown no evidence of any benefit for the combination [30, 78]. This could well be attributed to the lack of standardized protocols for dosage and schedules for these epigenetic drugs as much as our limited knowledge of their mutual interaction. Future work will therefore need to be done at the molecular and the clinical level to avoid any undesirable effects of this type of treatments.

Additionally, both writer- and reader histone-modifying enzymes have also proved to be promising targets in clinical oncology. Bromodomain and extra-terminal motif (BET) proteins are readers that recognize acetylated lysine. BET inhibitors (iBETs) represent an emerging class of epidrugs that have shown anti-cancer activity in preclinical studies and are now in late-stage clinical trials [79] (Fig. 3). iBETs reversibly bind to the bromodomain of BET proteins and disrupt critical protein-histone interactions. BRD4, which is translocated in some cancers, is crucial for the expression of oncogenes such MYC, and the pro-inflammatory gene NFKB is one of the better-studied targets of iBET small molecules [66]. Similar to iDNMTs and iHDACs, iBET can be considered as a broad reprogrammer drug, causing large-scale changes in gene expression [30]. It is well accepted that epigenetic inactivation of drug sensitivity genes is one of the causes of the complex process by which tumor cells acquire drug resistance to cytotoxic drugs. The hypothesis is that reactivation of these genes by the epigenetic drugs will sensitize the tumors to chemotherapy. Promising clinical data on a number of malignancies, including chronic leukemia and colorectal, ovarian, lung, and breast cancer, are robustly demonstrating that epigenetic therapy has the potential to overcome chemotherapy resistance and re-sensitize cancer cells to previously ineffective therapies [80]. Additionally, iHDACs and iDNMTs can increase sensitivity to DNA binding chemicals, such as cisplatin and doxorubicin (DOX), by influencing chromatin status, and thus chromatin accessibility to these drugs. Opened chromatin incorporates these drugs more easily than compact chromatin, resulting in an implementing efficacy of the drug to kill cancer cells. A similar correlation also applies to irradiation [81].

Epigenetic therapy may lead to sensitization not only to standard chemotherapy, but also to new emerging immunotherapy strategies. Many cancer cells acquire immune evasive phenotypes that render them “invisible” to the immune system. One of the rationales for using immunotherapy in combination with epigenetic drugs is that cancer cells can employ epigenetic silencing to hide from the immune system by shutting off the expression of certain cell surface molecules that play a crucial role in the efficient recognition and elimination of “intruders” by the immune system. It has been demonstrated that iDNMTs and iHDACs can reverse immune escape via several mechanisms, such as enhancing the expression of tumor-associated antigens or/and other immune-related genes [82]. Currently, ongoing clinical trials are evaluating combinations of epigenetic drugs and immunotherapy against many cancer types, including leukemias, metastatic melanoma, metastatic kidney cancer, peripheral neuroectodermal tumors, non-small cell lung cancer, and metastatic colorectal cancer [83].

## Epigenetics in precision medicine

Cancer is an extremely complex disease characterized by extensive inter- and intratumor heterogeneity. What we consider as a unique clinical disease or cancer subtype can actually in itself present great differences in important tumor-associated hallmarks. Furthermore, spatial and temporal clonal diversity within the same tumor can be observed at the molecular level. Such cancer heterogenicity may influence responsiveness to therapies and also, in part, explain the failure of some current cancer therapies, given that they are designed to treat all patients with a standard treatment, which does not take into account the unique profile of each individual patient. That is, the current regimens of cancer treatments, including surgery, chemotherapy, and radiotherapy, are not selective and can generate many side effects. Surgery cannot be applied to all kinds of cancer and is linked with a high risk of recurrence. The radiation and chemotherapy approaches are based on killing cancerous cells, but inevitably, they also attack healthy cells and induce non-target tissue toxicity and, very frequently in the case of chemotherapy, the onset of drug resistance. Moreover, radiation has been found to induce stemness in cancer cells, resulting in the enrichment of the cancer stem cell (CSC) subpopulation with increased resistance to radiotherapy [84].

At present, the challenge is to design new anti-cancer drugs conjugated with cancer-specific biomarkers and to select a site-specific delivery system that is able to optimize treatment efficacy and minimize toxicity.

“Omics” technologies are advancing our understanding of cancer genetics and epigenetics, allowing the delineation and definition of specific cancer subtypes and the identification of patient-specific biomarkers which advances personalized-targeted therapy. To date, there has been some success in precision therapy, particularly in the use of tyrosine kinase inhibitors such as imatinib, gefitinib, cetuximab, or trastuzumab, among others [85, 86].

Epigenetic cancer biomarkers largely focus on DNA methylation, mainly because histone marks are less stable modifications and present more technical challenges [44]. One of the best-characterized epigenetic markers is the hypermethylation of the glutathione S-transferase (GSTP1) gene in the biological fluids of prostate cancer patients [42, 44, 45]. Hypermethylation of APC, RASSF1, PTG2, and MDR1 has also been observed in prostate cancer samples, and the combination of these markers with GSTP1 has been demonstrated to be capable of reliably distinguishing between primary cancer and benign tissue with high sensitivity and specificity [87].

Epigenetic biomarkers can be useful to predict therapeutic drug response [42, 44, 45]. Examples include the association between hypermethylation of BRCA1 and increased sensitivity to platinum-based chemotherapy in ovarian and breast cancer [88], the correlation between GSTP1 methylation and the response to doxorubicin treatment [89], and the better response observed in glioblastoma treated with alkylating neoplastic agents associated with MGMT promoter methylation [90].

Although an ever-growing number of epigenetic biomarkers are emerging, and their reliability has been proven in terms of reproducibility and accuracy [91], none has yet been approved for clinical use. One of the limitations is that epigenetic variations can suffer from the direction of causality problems, since not all the epigenetic changes are functional, but may rather be induced by external factors [73]. The fact that chemotherapy and radiotherapy are still the first line choices for cancer treatment even though they can act as potent epigenetic modulators, and the fact that epigenetic modifications are cell-specific and can be directly impacted by environment and aging, are all variables that need to be taken into account when considering epigenetic modifications as possible cancer biomarker. In spite of this, diagnostic tools such as the EPICUP test, mentioned in the previous section, show the potential of epigenetic marks in general, and DNA methylation in particular, in the clinical management of cancer patients [33, 45].

Additionally, the growing number of epimutations identified in various cancers may lead to the development of targeted therapies to treat specific patient subtypes, based on the presence of specific mutations in epigenetic pathways. Drugs designed to inhibit the activation of mutations in IHD and EZH2 have entered early-stage clinical trials for the treatment of multiple types of hematological malignancies and genetically defined solid tumors. Pinometostat (EPZ-5676) is a small molecule inhibitor of the DOT1L enzyme, a histone methyltransferase that methylates lysine 79 of histone H3. The translocations involving the mixed lineage leukemia (*MLL*) gene at chromosome locus 11q23 generally define a subset of patients with AML or acute lymphoblastic leukemia (ALL) with poor prognosis. This translocation results in the recruitment of DOT1L to aberrant target sites with the subsequent methylation of H3K79 and gene transcription at the loci associated with hematopoietic transformation, including *HOXA9* and *MEIS1* [92]*.* Treatment of MLL-rearranged cells with pinometostat reduces histone 3 lysine 79 methylation (H3K79me2), decreases MLL target gene expression, and selectively kills leukemia cells. The potential for targeting DOT1L in MLL-rearranged leukemia is currently undergoing clinical assessment [93]. In summary, new therapies that specifically target epigenetic writers and readers are emerging, and they have the potential to be used for personalized cancer medicine.

## Nanomedicine and its effects on the epigenome

Advancements in cancer treatments are coming not only from new targeted therapies, but also from new site-specific delivery systems, which aim to carry high doses of a drug to the target site with minimum harm to healthy tissue.

The growing interest in applying nanotechnology to cancer, known as nano-oncology, is mainly the result of the possibility of creating and/or manipulating materials at the nanoscale (particles with dimension ranging between 1 and 100 nm) with tailored properties.

Nanotechnology-based agents have the potential to overcome many of the side effects caused by conventional cancer treatments, and in addition to drug delivery, nanoparticles (NPs) represent a useful tool for improving early detection and discovering new biomarkers and in imaging, as well as in cancer immunotherapy [94, 95]. NPs currently in use include biodegradable polymeric nanoparticles, dendrimers, solid lipid nanoparticles, liposomes, inorganic materials (e.g., metal nanoparticles, quantum dots), and biological materials (e.g., viral nanoparticles, albumin nanoparticles). Several NP platforms have been approved for cancer treatment, and several more are at present under clinical investigations [96]. NPs have the potential to overcome some of the limitations of conventional drug delivery systems, such as enhancing the pharmaceutical properties of the molecules (e.g., stability, solubility, circulating half-time, and tumor accumulation), minimizing non-specific distribution, and allowing for specific cancer targeting, thus preventing undesirable off-target and side effects in addition to improving intracellular penetration and overcoming drug resistance.

The preferential accumulation of NPs in tumors is generally ascribed to their enhanced permeability and retention effect (EPR). Sustained angiogenesis is a hallmark of cancer, and these newly formed tumor vessels are usually abnormal in both form and architecture. This resulting state of leaky and disorganized tumor vasculature enables NPs to preferentially enter the tumor interstitial space, while the lack of effective lymphatic drainage causes their retention, and consequent accumulation, in tumor tissue [97]. Several studies have confirmed the preferential accumulation and extended retention of NPs compared with uncoated drugs [98]. EPR is also known to differ greatly between tumor types and patients, as well as varying over time for the same patient and being influenced by treatment regime, and for this reason, it cannot be considered as a general feature of all cancer [99]. A recent study analyzing clinical data related to nanomedicine, including the magnitude of the EPR effect, has shown that it depends on tumor types and size [100]. In particular, pancreatic, colon, breast, and stomach cancers showed the highest levels of accumulation of nanocarriers containing drugs or imaging agents, and large-sized tumors had a higher accumulation than either medium- or very large-sized tumors. Moreover, these researchers found that other factors, such as tumor perfusion, angiogenesis, and inflammation in tumor tissues, coupled with patient-to-patient variations, also eventually affected the extent of the EPR effect.

These observations underline the importance of stratifying the subpopulations of cancer patients based on their likelihood of accumulating NPs through the EPR effect [96]. Several nanodrugs, mainly liposome-based NPs, have been developed and USFDA approved, both as single agents or in combination therapy (see review [101]).

When an NP enters a biological environment, several proteins bind to its surface, leading to the formation of a “corona.” These interactions can modify the physicochemical properties of NPs and, in turn, determine the physiological response they elicit, such as cellular uptake, distribution bioavailability, and toxicity, and thus effectively generating a particle with a “new biological identity” [102]. The binding of opsonin (opsonization) represents one of the biggest biological barriers for NP bioavailability as it triggers immune system recognition and clearance by mononuclear phagocyte system (MPS). In fact, to take advantage of the EPR effect, NPs need to circulate for a prolonged period. In order to circumvent this limitation and prolong the circulation time of NPs, various methods have been developed to mask NPs from MPS. One of the most preferred is to coat the NP surface with polyethylene glycol (PEG) chains [103], and many alternative strategies have advantages over the PEG system in terms of increasing the blood circulation time of NPs, such as the conjugation of self-markers like CD47 on the surface of NPs or camouflage the NP surface with cellular membranes purified from leukocytes, erythrocyte, and thrombocytes [96].

Although long circulation times allow for the effective transport of NPs to the tumor site through the EPR effect, successful internalization also has an important role in enhancing NP retention and therapeutic efficacy. One strategy to increase both cellular uptake and the specificity of drug delivery is by active NP targeting approaches. Different events contributing to carcinogenesis, such as angiogenesis, uncontrolled cell proliferation, tumor, and metastatic microenvironment, can act as the target for NP systems. The most common approach is to conjugate NPs with targeting ligands that selectively recognize tumor antigens, carbohydrate-like structures, or growth factor receptors, all of which are usually overexpressed in tumor cells. This ligand-mediated interaction is then followed by internalization and the intracellular delivery and accumulation of the payload drug. A variety of tumor-targeting ligands have been identified, such as antibodies, polysaccharides, peptides, transferrins, folates, and other small molecules [104].

In order to be functional, modified NPs need to effectively reach the tumor cell surface after extravasation across the vasculature endothelium. However, only small fractions of intravenously administered modified NPs are able to reach the target site, limiting the efficacy of active targeting strategies. Tumor heterogeneity and the ability of cancer cells to adapt over time to external stimuli represent additional barriers for the clinical achievement of the nanotherapies discussed here. To circumvent some of these limitations, general features of cancer rather than tumor-specific markers could be exploited for targeting approaches. Tumor vasculature is crucial for tumor growth and metastasis. Cell-specific targeting can be directed towards tumor vasculature by coating NPs with ligands that specifically bind to overexpressed receptors, such as vascular endothelial growth factor (VEGF), integrin, and matrix metalloproteinase, on the surface of tumor endothelial cells [105]. The advantage of targeting tumor vasculature rather than cancer cells is that this strategy can be generalized to almost all solid tumors since they all depend on angiogenesis and the fact that tumor endothelial cells are directly exposed to circulating blood, which can greatly facilitate the binding of functionalized NPs [106].

Both internal and external stimuli can trigger the release of drugs by generating a change in the structure of the nanocarrier. Hypoxia, the condition in which proliferating tumor cells are deprived of oxygen, is a common feature of solid cancers and plays a critical role in chemoresistance, radioresistance, invasiveness, altered metabolism, and genomic instability. Hypoxia-responsive polymeric NPs have been engineered to selectively release hydrophobic agents under hypoxic conditions, which results in the accumulation of NPs at the hypoxic regions of tumor cells [107]. pH-sensitive NP systems have also been developed, and they target extracellular acidification, a condition typical not only of tumor microenvironments, but also of several diseases as ischemia, inflammation, arthritis, and atherosclerosis. The key principle is to generate NPs that are stable at physiological pH but destabilized upon acidification following cellular internalization, leading to the consequent release of the uploaded drug in the cytosol [108]. Another avenue is the development of stimuli-responsive drug carriers such as temperature-, magnetic-, and ultrasound-sensitive NPs which aim to modulate drug release rate and site specificity [109].

With the now well-established concept of combination therapies, NP formulations have been exploited for the synchronized co-delivery of two drugs in a single particle in order to synergize their activities. Multifunctional NPs that co-deliver a therapeutic agent alongside a diagnostic or tracking agent provide a great platform for integrating diagnostics, therapy, and follow-up data. In fact, this theranostic approach could enable real-time monitoring of the fate of an NP and the concomitant effects of the drugs it is carrying on tumoral tissue. Multifunctional NPs could have a great impact on personalized medicine as they have the potential to provide direct clinical data that can assist in determining whether selected patients could receive therapeutic benefit from NP-based therapy. This could be particularly important in terms of selecting a subpopulation of patients that could benefit from a given NP treatment on the basis of the EPR effect.

Although it is clear that nanomaterials are promising tools for cancer treatment and diagnosis, many challenges still need to be overcome before their clinical use. Physiochemical parameters are crucial for the therapeutic efficacy of NPs. However, preclinical evaluations, using both in vitro and in vivo animal models, do not always effectively reproduce the complexity of human tumors, thereby explaining, at least in part, the translational gap observed between preclinical studies and the outcomes of clinical trials.

Despite their obvious clinical potential, the accumulation of NPs in the body still raises concerns about their safety for human health [110–112]. Nanomaterials can induce genotoxicity, directly by their interaction with genetic material or indirectly via intermediate biomolecules that cause DNA injury or chromosomal abnormalities. Indirect genotoxicity is principally due to oxidative stress induced by the induction of reactive oxygen species (ROS) by NPs and is strongly linked to inflammatory cell response and immunotoxicity [111]. Moreover, numerous studies have demonstrated the possible epigenetic toxicity of NPs [110–112]. In fact, mammalian cells exposed to nanomaterials exhibit changes in global as well as locus-specific DNA methylation, histone post-translational modifications, and ncRNAs expression [112, 113].

**Fig. 4:**
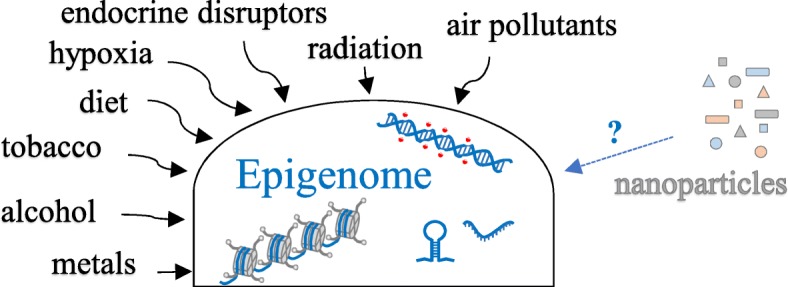
External factors that affect the epigenome. Although the associations between exposure to various external factors and alterations in the human epigenome, with consequent risk to health, have been demonstrated, in the case of nanoparticles, these associations have not been well studied

Despite the growing evidence supporting the epigenetic effect of NP exposure, the potential health risk posed, especially in terms of nanomedicine, continues to be debated (Fig. 4). One limitation is that epigenetic toxicity is mostly demonstrated in in vitro and animal models, which, as mentioned earlier, does not always adequately reproduce human complexity. Additionally, the long-term effects of NPs on epigenetic modifications need to be elucidated in order to distinguish between the physiological response of the cell to NP exposure and pathological changes. Another important aspect to take into account is the individual genetic variability in susceptibility to exposure to specific NPs [112, 114].

The challenge for the future will be to integrate all these aspects in a complete picture which considers both genotoxicity and epigenetic toxicity in the assessment of the safety of NPs.

## Epigenetic approaches in nanomedicine

Despite the ever-growing advances, epigenetic medicine still faces numerous challenges. As mentioned above, current USFDA-approved epigenetic drugs lack locus specificity and are not selective in inhibiting different DNMTs and HDAC isozymes. This lack of specificity induces unintended off-target effects, with consequently high drug toxicity and the failure to induce long-term response [73, 115]. Additionally, the low solubility and permeability of these epigenetic drugs and their poor pharmacokinetic properties such as lack of stability and bioavailability represent significant drawbacks for their broader clinical application. It is therefore of major importance to refine target specificity and improve drug stability and delivery efficiency in order to fully exploit the clinical potential of these drugs. Nanoscale delivery systems and prodrugs have the potential to overcome some of the clinical issues of the current epigenetic drugs, as they can protect against premature hydrolysis, increase bioavailability, and enhance cellular internalization and tumor-targeted delivery.

In order to address the stability and tolerability issues, second-generation nucleoside analogs (demethylating agents) are currently being tested (Fig. 3). Guadecitabine (SGI-110), a dinucleotide prodrug containing 5-aza-CdR, was rationally designed to be resistant to cytidine deaminase, ensuring a longer circulating half-life in patients and the prolonged exposure of tumor cells to the active metabolite. SGI-110 is being tested in clinical trials for MDS, AML, ovarian cancer, and hepatocellular carcinoma HCC, both as a single agent and in combination therapies [116]. RX-3117 is an orally available cytidine analog, currently being investigated in phase I/II clinical trial in solid tumors. Apart from exerting its cytotoxic effects through the inhibition of DNA and RNA synthesis, RX-3117 also has a potent anti-tumor activity which works through the dose-dependent inhibition of DNMT1 [117]. CP-4200 is a prodrug developed by conjugating azacytidine molecule with a fatty acid, elaidic acid. It was designed to decrease the dependency of drugs on the conventional nucleoside transporters involved in azacytidine uptake. Preclinical studies have demonstrated that CP-4200 has a significantly increased and stronger epigenetic modulatory effect than its analog 5-azacytidine in several human cancer cell lines [118]. Although further preclinical studies are in progress, yet no clinical trials have started. Other promising prodrugs of decitabine and azacytidine such as NPEOC-DAC and 2′3′5′triacetyl-5-azacytidine are also currently in the preclinical trial phase [119].

**Fig. 5:**
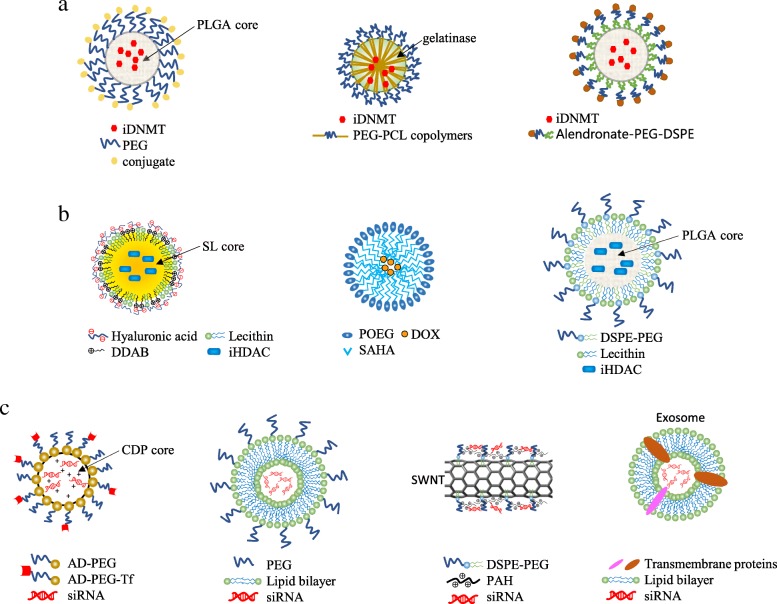
Schematic representation of the different types of nanocarriers for epigenetic therapy in cancer. **a** Vehicles for transport and delivery of iDNMTs in poly(lactic-co-glycolic acid) (PLGA)- and poly(ethylene glycol) (PEG)-based nanomicelles, in gelatinase with PEG and poly-ε-caprolactone (PCL), and in alendronate-PEG-2-distearoyl-sn-glycero-3-phosphoethanolamine (DSPE). **b** Vehicles for transport and delivery of iHDACs in hyaluronic acid (HA)-coated cationic solid lipid (SL) nanoparticles (didecyldimethyl ammonium bromide (DDAB) is used as cationic lipid), in micelles based on SAHA-based prodrug polymer (POEG-b-PSAHA) (POEG: poly(oligo(ethylene glycol) methacrylate)), and in a PLGA-lecithin-PEG core-shell system (DSPE: 1,2-dioctadecanoyl-sn-glycero-3-phosphoethanolamine). **c** Vehicles for transport and delivery of siRNA in cationic cyclodextrin-based polymer (CDP) modified with a terminal adamantane group (AD-PEG) and some AD-PEG conjugated to human transferrin (Th), in PEGylated liposomes, in single-walled nanotubes (SWNTs) functionalized with PEG-DSPE and polymer poly(allylamine hydrochloride) (PAH), and in exosome-based systems

Nanoscale delivery systems can also improve the therapeutic efficacy of demethylating agents (Fig. 5a). NP delivery systems can be developed so that they protect demethylating agents from degradation, increase permeability and enhance targeting specificity, and minimize adverse effects. Several synthetic or natural biodegradable materials can be used for the delivery of demethylation agents. A nanodelivery system which uses PLGA-PEG di-block has been formulated to stabilize the conjugation of AZA (Fig. 5a). In murine xenograft models of breast cancer, the conjugated form of AZA showed enhanced therapeutic efficacy compared to free AZA, including increased drug solubility and bioavailability, enrichment in cancer cells, pH-sensitive drug release, and a greater anti-proliferative effect [120].

It has been reported that lipid nanocarriers can protect a drug from acidic degradation, increase intestinal permeability, and promote oral absorption, for example, there is promising evidence to support the use of a nanostructured lipid carrier for the oral delivery of decitabine [121]. Another medium, nanogels (NGs), can be designed to release their encapsulated drug in response to the patient’s physiological environment or specific conditions such as temperature, pH, or molecular recognition [122]. Decitabine-loaded NG has been used to circumvent drug resistance mechanisms in cancer cell lines [123] (Fig. 5a), and a combination of epigenetic drugs (DAC + SAHA) encapsulated in biodegradable NGs was found to more effectively overcome drug resistance than the same drugs in solution. NG-mediated delivery has been shown to result in the greater stability of drugs, better cellular uptake, and more sustained effects of drugs compared to unmodified drugs, and thus enhanced efficacy [124]. Intelligent NPs, comprising gelatinase with polyethylene glycol (PEG) and poly-ε-caprolactone (PCL), have been used to deliver DAC and have shown promising results in overcoming epigenetic-mediated multidrug resistance in both in vitro and in vivo models [125].

Combining NPs loaded with epigenetic-targeted and a chemotherapy drug is emerging as a promising strategy to achieve greater therapeutic benefits and reduce side effects. A combination of DAC and arsenic trioxide (ATO) has been demonstrated to act synergistically as an MDS treatment. The co-packaging of DAC and ATO into alendronate-conjugated bone-targeting NPs (BTNPs) combines the advantages of both polymeric NPs and liposomes simultaneously in order to obtain the controlled and targeted release of the co-delivered drugs. BTNPs consist of a poly (d, l-lactide-co-glycolide)-cholesterol polymer that releases the drugs in a controlled fashion, within a shell made of alendronate-PEG-lipid which is designed to target the bone marrow [126] (Fig. 5a).

Erythrocytes have been proposed as safe and biocompatible carriers especially for agents that show limited tissue penetration or are rapidly inactivated. One example is erythro-magneto-hemagglutinin virosomes (EMHVs), an erythrocyte-based drug delivery system, that combines super-paramagnetic NPs with hemagglutinin fusion protein, which has been used as a carrier for 5-aza-2′-dC [127]. The magnetic nature of this delivery system allows the drugs to be targeted towards the specific tissue by applying an external magnetic field, while the presence of the fusogenic glycoprotein on the EMHV membrane mediates the drug’s efficient intracellular release [128]. In prostate cancer xenograft mouse models, using an EMHV delivery system significantly improved the pharmacokinetics/pharmacodynamics of decitabine and induced a significant reduction in tumor mass at much lower concentrations than its usual therapeutic dose [128].

As for iDNMTs, a long list of iHDAC prodrugs is emerging which have the potential to improve the pharmacokinetics and pharmacodynamics of these drugs and augment their efficacy, especially in the treatment of solid tumors (see review [115]). Loading iHDACs onto NPs is, however, complicated by the low solubility of the compounds, and therefore, currently, such inhibitors need to be converted into soluble prodrugs during the preparation of the NP vector [129] (Fig. 5b).

Another drug delivery system, based on endocytosis-mediated internalization, takes advantage of the initial moderately acidic pH of the early stages of endosome formation. Thus, pH-responsive prodrugs have been developed which are not released at physiological pH during blood circulation, and delivery is instead triggered intracellular following endocytosis [130]. Such pH-responsive prodrugs can be conjugated to an NP which is able to enter cancer cells and thus protecting it from external metabolism. Such modified delivery systems can dramatically improve the efficacy of iHDACs, in particular in solid tumor therapies, by decreasing the side effects [131].

The clinical efficacy of the USFDA-approved iHDAC VOR is limited by its poor aqueous solubility and low permeability, thus limiting its bioavailability in the systemic circulation. Numerous studies have demonstrated that VOR delivery using solid lipid NPs could significantly improve the chemotherapeutic potential of this drug [132]. In addition to improving the drug’s pharmacokinetics and efficacy, VOR solid lipid NPs have been demonstrated to overcome multidrug resistance in P-glycoprotein-overexpressing cells [133]. Solid lipid NPs can be furthermore modified with a targeting moiety in order to increase their targeting ability and cellular internalization. Hyaluronic acid (HA)-based nanomaterials have been extensively used as active targeting delivery systems for cancer therapy (Fig. 5b). Many cancer cells, including tumor-initiating stem cells, are known to overexpress the HA-binding receptor CD44, and HA has often been modified with a drug carrier in attempts to improve drug delivery to CD44-overexpressing cancer cells [134]. HA-coated cationic solid lipid NPs have been successfully used in animal models for the tumor-targeted delivery of VOR. This delivery system enables the slower release and faster penetration of VOR in CD44-overexpressing cells, leading to reductions in the toxicity impact on normal cells. Additionally, the negatively charged surface allows the VOR to circulate longer in the blood, thereby increasing the chance of it reaching the tumor area [135]. Although iHDACs have significant anticancer activity as a monotherapy in hematological malignancies, they have been shown to have limited clinical benefit in solid tumor in this form. iHDACs have, however, been used successfully in conjunction with other anticancer treatments such as chemotherapy and radiotherapy [136, 137].

Carrier-mediated combination therapy can offer many advantages, particularly with respect to the synchronization and control of the pharmacokinetics and appropriate dosage of each drug. For example, SAHA has been modified to produce a cleavable amphiphilic SAHA-based prodrug polymer, POEG-b-PSAHA, which retains the pharmacological activity of SAHA and is effective in formulating DOX (Fig. 5b). In a syngeneic breast cancer model, DOX-loaded POEG-b-PSAHA led to an improved therapeutic index, suggesting that SAHA-based prodrug micelles can serve as a dual functional carrier for combination strategies [138].

Preclinical studies have indicated that a number of iHDACs can sensitize tumor cells to radiotherapy in a variety of solid malignancies. Second-generation iHDACs, such as quisinostat, have shown more potent and prolonged activity as compared with first-generation drugs. Unfortunately, this may also translate into the enhanced sensitization of both tumor and normal cells to the effects of radiotherapy, resulting in an increased toxicity. In order to circumvent these limitations, PLGA NP formulations of first-generation iHDAC SAHA and second-generation iHDAC quisinostat have been designed which have been shown to enhance the response of tumor cells to radiation (Fig. 5b). This is mainly due to the fact that NP iHDACs preferentially accumulate in tumors and can release their load in a slower and more controlled fashion, facilitating their synchronistic interaction with radiotherapy [139].

NP formulations have also been expanded to other classes of iHDACs in order to achieve a higher therapeutic index and reduce off-target toxicity. Preclinical studies are demonstrating how epigenetic drugs could benefit from more appropriate delivery systems that would enable them to more fully exploit their therapeutic potential. However, epigenetic drug delivery methods using NPs is still at a relatively early stage of development due to its restricted application in mainly in vitro and animal models, the results of which are still far from being translated into in vivo settings.

Clinical trials are more advanced in terms of using NPs for the successful delivery of antisense oligonucleotides and siRNA/miRNA, either alone or in combination with chemotherapeutic agents. RNAi-based medicine involves the delivery of double-stranded siRNA or miRNA to silence target genes with high specificity and efficacy. This therapy can also be directed towards and act against non-druggable targets in cancerous cells since, due to their inaccessibility or their lack of enzymatic activity, not all the proteins deregulated in cancers are targetable with conventional molecules. Although very promising, this method faces several limitations, such as a lack of stability against extracellular and intracellular degradation by nucleases, an inability to cross cell membranes; considerable off-target effects; and the stimulation of the innate immune system [140].

NP-based delivery systems are an effective solution to overcome these limitations, and promising results have come from recent cancer RNAi trials. Examples of successful active targeting have been demonstrated with nanocarriers conjugated with an anti-transferrin receptor single-chain antibody fragment designed to target cancer cells by binding to the transferrin receptor (TfR) [141, 142]. One such is SGT-53, a transferrin receptor-targeted liposomal nanocarrier which encapsulates a normal human wild-type p53 DNA sequence in a plasmid backbone, and is currently in phase II trials for the use in the treatment of recurrent glioma and metastatic pancreatic cancer. This system has shown a very high ability to target tumor cells with high specificity and produce a significant amount of antitumor activity. This is important because a variety of tumor types treated with SGT-53 have been shown to be sensitized to conventional radiation/chemotherapy [143]. CALAA-01 is another transferrin receptor-targeted NP encapsulating siRNA which acts against the M2 subunit of ribonucleotide reductase (RRM2), and it has shown potential antineoplastic activity in phase I trials which involve patients with a variety of cancers [144] (Fig. 5c).

Several examples of liposome-based RNAi delivery systems have entered the advanced stages of human trials, notably siRNA-EPHA2-DOPC (siRNA against EPHA2), MRX34 (miR-34), Atu027 (siRNA against protein kinase N3), and PNT2258 (DNA oligonucleotide against BCL-2). An optimized formulation of lipid NPs (LNPs) is commonly used as nanoparticle-based delivery vehicles for RNA (Fig. 5c). Promising phase I/II clinical trial has been conducted for TKM-080301 (LNP formulation of an siRNA against PLK1), ALN-VSP (LNP siRNA against KSP and VEGFA), DCR-MYC (LPN siRNA against the oncogene c-Myc), and pbi-shRNA STMN1 lipoplex (LPN short hairpin RNAs against human stathmin 1 (STMN1) [96, 140].

The progress made in nanotechnology has also allowed the development of nanocarriers based on inorganic nanoparticles for the transport and delivery of siRNAs [145]. Nanocarriers based on single-walled carbon nanotubes (SWNTs) are of special interest due to the ease with which they can be loaded with biomolecules and their capacity to cross the cell membrane. SWNTs can be functionalized by their association with a lipopolymer composed of phospholipid 1,2-distearoyl-sn-glycero-3-phosphoethanolamine and poly(ethylene glycol) (DSPE-PEG) and then used as carriers of siRNAs for cancer gene therapy [146–148] (Fig. 5c).

In addition to the above, exosomes and self-assembled nucleic acid NPs are emerging as potential vectors with encouraging characteristics for clinical translation [149–151] (Fig. 5c). Although several clinical trials have highlighted the feasibility of NP delivery system for targeting siRNAs in tumors and investigating their therapeutic potential in cancer treatment, continued work is needed in order to increase potency, and the pharmacokinetic and biocompatibility profiles of existing delivery carriers [151].

## Conclusions and future directions

Many advances have been made in the identification of epigenetic alterations in cancer and their associations with the functional changes that can induce and/or enhance the tumorigenic process. The identification of these epigenetic biomarkers has been useful for precision medicine in terms of diagnosis, prognosis, and even the evaluation of response to treatment. However, the use of epigenetic drugs has been limited, due, in particular, to their low specificity and the appearance of associated pleiotropic effects. The emergence of new generation technologies has allowed, on the one hand, the identification of specific epigenetic alterations related to individual tumor subtypes and, on the other, the development of more stable and effective epigenetic drugs. Additionally, the appearance of nanotechnology in medicine, and specifically the development of nanosystems for the transport and target-specific release of epigenetic drugs, will help to advance personalized-targeted therapy.

There do, however, remain many challenges for the future. One of these will be to study the complete epigenome of tumor cells at the single cell level, which will allow us to not only characterize all the epigenetic alterations associated with a particular tumor, but also to identify the specific cells that systems can be designed to target. Other challenges include testing the efficacy in humans of the new generation of epigenetic drugs either alone or in combination with other drugs, the design of systems to monitor responses to antitumor therapy, and determining the effect on the epigenome of the accumulation of nanoparticles used in some systems of encapsulation, transport and release of this type of antitumor drug.
